# ‘A world of competing sorrows’: A mixed methods analysis of media reports of children with cancer abandoning conventional treatment

**DOI:** 10.1371/journal.pone.0209738

**Published:** 2018-12-21

**Authors:** Caroline Diorio, Michael Afanasiev, Kristen Salena, Stacey Marjerrison

**Affiliations:** 1 McMaster Children’s Hospital, Hamilton, Canada; 2 Children’s Hospital of Philadelphia, Philadelphia, Pennsylvania, United States of America; 3 Institute of Geophysics, ETH Zurich, Zurich, Switzerland; 4 Faculty of Health Sciences, McMaster University, Hamilton, Canada; Universidad de Navarra, SPAIN

## Abstract

**Background:**

We aimed to provide health practitioners greater insight into the public perception of traditional and complementary medicine (T&CM) use. Our objectives were to identify news media reports of children abandoning conventional treatment for traditional and complementary medicine, analyze the thematic content of these news articles and estimate the tonality portrayed.

**Methods:**

LexisNexis and Factiva were searched for terms related to cancer, children and T&CM. Inclusion criteria were children less than 18 years, in curative phase of treatment who attempted to abandon conventional therapy for any traditional and complementary medicine use. A secondary search was performed in LexisNexis, Factiva and Google News Archive with the names of children in identified cases. Qualitative analysis of news media reports was completed using a grounded theory approach. Quantitative analysis of article sentiment was performed using a linear support vector machine.

**Results:**

Seventeen cases occurring between 2002 and 2016 were included. Five main themes were identified: treatment as torture, power imbalances, rights of parents, evidence versus beliefs and the rights of Indigenous Peoples. Sentiment analysis revealed an overall negative tone, as demonstrated by 73% of the articles.

**Interpretation:**

A better understanding of factors that lead to abandonment of conventional therapy for traditional and complementary medicine as portrayed in the news media may help healthcare providers prevent the occurrence of these cases.

## Introduction

Traditional and complementary medicine (T&CM) use among children with cancer is common worldwide, with use often motivated by intention to cure.[1, 2] These T&CM therapies include any health practices outside of the dominant, Western or allopathic paradigm, hereafter referred to as conventional therapy (CT). With regard to cancer treatments, CT typically includes chemotherapy, radiation and/or surgery. Treatment abandonment, defined as failure to begin or complete curative CT,[3] may be related to a desire for exclusive use of T&CM.[4] Treatment abandonment is rare in high-income countries (HIC), with estimates of less than 5% of families attempting to abandon treatment.[5] A recent study in adult cancer patients showed that exclusive use of T&CM strategies without CT is associated with a greater risk of death.[6]

There is a paucity of medical literature examining why some families prefer T&CM over CT. However in HIC, cases where families leave or attempt to leave CT may be published in the news media. These cases may have a disproportionate impact on public perception of the interplay between T&CM and CT. Previous studies have examined legal cases where families have refused CT in HIC. [7, 8] To our knowledge, no studies have examined media reports of abandonment of CT for T&CM in HIC.

We reviewed media portrayals of families leaving CT for T&CM in pediatric cancer in order to help practitioners understand what pre-conceived notions exist about the role of T&CM and CT, with the hope of improving collaboration between healthcare providers, T&CM practitioners and families.

## Methods

### Data sources and searches

We searched the news databases LexisNexis and Factiva for terms related to cancer, children and traditional and complementary medicine (T&CM) (see S1 Appendix) between May 24, 2006 and May 24, 2016. LexisNexis is an online database of over 350 full text newspapers from Europe and Norther America.[9] Factiva is an online database from Dow Jones containing over 32, 000 sources. Both databases include local, national and international news sources.[10] For feasibility, we reviewed only news articles in English and over a ten-year period; however, included any cases that had occurred since 2002, the year when the first iteration of the World Health Organization (WHO) T&CM strategy was published.[11, 12] Articles were screened by two reviewers (CD and SM) and cases were included based on the following criteria: 1) Child (<18) with a cancer diagnosis, 2) Attempt was made to abandon or successfully abandoned cancer therapy that was deemed lifesaving or necessary by the treating team, 3) Case occurred between January 1, 2002 and May 24, 2016 and 4) Treatment was left to seek out any type of T&CM as per National Center for Complementary and Integrative Health (NCCIH) definition, including prayer.[13] Exclusion criteria included: 1) Adult (>18) patient, 2) Non-cancer diagnosis, 3) Child in palliative phase of treatment and 4) Treatment sought not T&CM.

After cases were identified through the primary search, a secondary search was initiated using the child’s name. Where the child’s name was not available, specific terms related to their case were searched. Databases searched included LexisNexis Academic, Factiva and Google News Archive. All articles available through LexisNexis Academic and Factiva were retrieved for sentiment mining but only cases with greater than 15 articles were included in the sentiment analysis. A sample of up to 20 articles for each identified case were retrieved for qualitative analysis.

### Outcomes

For every case identified, we sought to identify the following outcomes where available: name, age, gender, location, conventional therapy received, T&CM proposed or used, and clinical outcome.

### Qualitative analysis

Two study team members (KS and CD) analyzed 20 articles for each case, or all articles that were available if less than 20 articles were published, from a grounded theory perspective.[14, 15] Documents were coded and concepts were identified based on coded elements. Concepts were compared between study team members and organized into themes and sub-themes using a consensus-based approach.

### Sentiment analysis

Articles related to 10 patients were analyzed (Abraham Cherrix, Cassandra Fortin, Daniel Hauser, Jessica Crank, JJ, Katie Wernecke, Makayla Sault, Neon Roberts, Oshin Strachan and Sarah Hershberger) using automated sentiment analysis. 100 articles from across the case spectrum were analyzed by two study team members (CD and KS), and each article’s sentiment was rated as positive or negative. Designation of sentiment was appended based on the overall tone of the article. We did not distinguish between articles that were negative or positive toward a specific party, i.e. the patient or the physician, but instead evaluated the overall tone of the article.

These articles, which composed the training dataset, were then loaded into a computer program and ‘vectorized’. In machine learning, ‘vectorization’ refers to the process whereby real data are transformed into a numerical representation more easily interpretable by computational algorithms. The vectorization of the training set followed the ‘bag of words’ approach. Each word was mapped to an index in a vector, with the numerical value of that vector component encoding the number of occurrences.[16] In order to reduce the number of unique features, each word was lemmatized and transformed into its base dictionary equivalent. Stop words, punctuation, digits, and proper nouns were removed. The lemmatization process was assisted by the use of the Natural Language Toolkit (NLTK) package for Python.[17]

Once lemmatized, the bag-of-words vectors were used as training data for a linear support vector machine (SVM). A SVM was chosen over other competing machine learning algorithms (i.e. neural networks) due to its relative stability in the presence of fewer samples.[16] The goal of a linear SVM is to find the maximum margin hyperplane between two classes—positive and negative articles—based on the components of each of the bag-of-words feature vectors and each vector’s associated class.

Several parameters were tuned in order to train the SVM. These included loss and penalty functions, weight of regularization parameters, and number of iterations required for stable convergence of a stochastic gradient descent (SGD) classifier. A 5-fold cross validation settled upon a modified huber loss function with an L2 penalty, a regularization weight of 0.001, and 10 iterations. When applied to the training dataset, the trained model classified approximately 70% of the documents correctly. All training and classification was done within the scikit-learn ecosystem, a Python package for machine learning and data analysis.[18]

## Results

### Outcomes

7722 articles were retrieved in the primary searches. After duplicates were removed, 6172 articles were screened by two reviewers (CD and SM). A total of 17 cases were identified that met inclusion criteria; features of the cases are outlined in Table 1.

**Table 1:** Cases of children abandoning conventional therapy for traditional and complementary medicine (T&CM) identified through primary and secondary searches.

Name, Age, Year	Diagnosis	Location	Conventional Therapy	T&CM Therapy	Case Details	Outcome
**Jessica Crank**, **14, 2002**	Ewing’s Sarcoma	Loudon County, Tennessee, USA	None received	Prayer (New Life Tabernacle Church)	Child developed large mass on her shoulder and was eventually taken to a walk-in clinic. Family failed to show up for pediatric oncology appointment and social services were involved. Child died without receiving therapy.	Died in 2003.
**Abraham Cherrix, 15, 2005**	Hodgkin’s lymphoma	Chincoteague, Virginia, USA	Chemotherapy	Hoxsey tonic, sugar free diet	Initially received chemotherapy for three months, declined further therapy. Child taken in to custody by child services and forced to undergo therapy. Family eventually reached an agreement with court for radiation and alternative therapy. As an adult, completed high dose therapy and autologous stem cell transplant therapy.	Alive as of 2017, post autologous stem cell transplant. “Abraham’s Law” enacted in Virginia, increased the rights of children aged 14 to 17 to refuse medical treatments.
**Katie Wernecke, 12, 2005**	Hodgkin’s lymphoma	Agua Dulce, Texas, USA	Chemotherapy	Prayer (Church of God), vitamins	Initially treated with chemotherapy but when physicians recommended radiation, parents refused prompting involvement of child services. Child taken in to protective custody. Cancer recurrence demonstrated, parents agreed to radiation and further chemotherapy.	Recurrence in 2007. Alive as of November 2011.
**Ryan Xiong, 4, 2006**	Acute leukemia (type not specified)	Anchorage, Alaska, USA	Chemotherapy	Shaman (Hmong tradition)	Child taken initially to shaman who recommended not seeking medical care. Eventually received treatment. Outcome unknown.	Received therapy. Alive as of 2006.
**Unnamed Boy, 11, 2008**	Relapsed acute lymphoblastic leukemia	Hamilton, Ontario, Canada	Chemotherapy	Oregano, green tea, chelation, vitamins, spiritual healing	Child relapsed. Father and stepmother wanted to abandon chemotherapy, child taken in to custody by CAS to complete chemotherapy. Custody subsequently returned to parents. Child also had FASD.	Child alive as of May 2014. Child diagnosed with PTSD and charged with arson, assault, mischief as a teenager.
**Daniel Hauser, 13, 2009**	Hodgkin’s lymphoma	Sleepy Eye, Minnesota, USA	Chemotherapy	Diet, supplements, herbs, water with acidic pH, acupuncture, acupressure, Nemenhah Band healing	Abandoned chemotherapy after one cycle. Court order to return to chemotherapy; mother fled with son to California. Arrest warrant issued. Mother returned voluntarily. Arrest warrant rescinded, custody returned to parents when they agreed to chemotherapy. Received chemotherapy.	Alive and well as of August 2011.
**Neon Roberts, 7, 2012**	Medulloblastoma	Brighton, United Kingdom	Surgery	“Natural remedies”	Mother took son into hiding rather than receive chemotherapy or radiation. Mother and child found, child placed in custody of father for full duration of treatment. Child received radiation and chemotherapy.	Alive and well as of January 2014.
**Sarah Parisian, 8, 2012**	Brain tumor (unspecified)	Minnetonka, Minnesota, USA	Surgery, proton therapy, chemotherapy	“Alternative medicine”	After one round of chemotherapy, family declined further. Physicians reported family to child services. Reached compromise with physicians and received less intensive chemotherapy.	Outcome unknown.
**Unnamed boy, 3, 2012**	Acute lymphoblastic leukemia	Singapore	Chemotherapy	Alternative therapy at Our Place International	Child services stepped in to have child remanded in to custody of hospital to receive chemotherapy. Parents eventually relented to therapy when child became ill.	Outcome unknown.
**Sarah Hershberger, 10, 2013**	Acute lymphoblastic lymphoma	Medina County, Ohio, USA	Chemotherapy	Alternative therapies in Central America, “God’s will”	Court appointed guardian when family tried to stop chemotherapy after 2^nd^ cycle. Family went in to hiding in Central America. Court and hospital eventually dropped case.	Child alive as of October 2015.
**Brayden O’Donohue, 9, 2013**	Acute myeloid leukemia	Newcastle, New South Wales, Australia	Chemotherapy, bone marrow transplant	Raw vegan diet, Chinese medicine, reiki, yoga and meditation	Parents reported to child protective services after balking at bone marrow transplant. No further action.	Alive following bone marrow transplant as of October 18, 2013.
**Aiden Pederson, 18 months, 2014**	Acute lymphoblastic leukemia	Ottawa, Ontario, Canada	Chemotherapy	Cannabis oil	Parents attempted to abandon chemotherapy after ten days. Mother agreed to chemotherapy and father lost custody of child.	Unknown.
**Makayla Sault, 11, 2014**	Acute lymphoblastic leukemia	New Credit First Nation, Ontario, Canada	Chemotherapy	Traditional Indigenous healing, treatment at Hippocrates Health Institute	Child wanted to stop chemotherapy after several cycles. Supported by her parents. Child services declined to intervene. No further chemotherapy.	Child died in January 2015.
**J.J., 11, 2014**	Acute lymphoblastic leukemia	Six Nations, Ontario, Canada	Chemotherapy	Traditional Indigenous healing, treatment at Hippocrates Health Institute	Mother refused chemotherapy in favour of alternative treatments. Child services involved, refused to intervene in case. Hospital took child services to court. Judge ruled that child’s right to traditional Indigenous medicine was protected by the Charter of Rights and Freedoms.	Child relapsed. Restarted chemotherapy at a different hospital. Alive as of May 2015.
**Cassandra (Callender) Fortin, 17, 2015**	Hodgkin’s disease	Hartford, Connecticut, USA	Chemotherapy	“Natural methods”	Child services took custody of child when she and her mother failed to arrive at medical appointments. Child ran away for a week, was subsequently kept in custody at a hospital.	Recurrence of disease in April 2016. Child of age at that time, recurrence treated with alternative therapies.
**Oshin Strachan, 6, 2015**	Medulloblastoma	Perth, Western Australia, Australia	Surgery	Alternative treatment in Asia. Nutrition focused therapy.	Parents refused chemotherapy and radiation. Mandated to receive chemotherapy by family court after a 3- month delay. No radiation.	Switched to palliative care in August 2016. Died in December of 2016.
**Unnamed boy, 10, 2015**	Osteosarcoma	London, United Kingdom	None	Traditional Chinese Medicine	Parents absconded with child to Poland. Judge pleaded for family to return for surgery.	Unknown.

### Qualitative analysis

Five main themes were identified. See Table 2 for a complete list of themes and sub-themes with supporting quotations.

1. Treatment as torture: the idea of cancer treatment being tantamount to torture was raised in discussion of many of the identified cases. For example, when describing the experience of their child Oshin receiving chemotherapy his parents stated, “It almost feels like Nazi Germany and I am honestly sickened by the treatment of all these children… nothing short of toxic hell”. [19]
2. Power imbalances: several reports portrayed the cases as a power struggle between conflicting decision makers, all trying to represent the best interest of the child. Parents were portrayed as feeling powerless in the face of medical systems: “Mr Strachan posted another comment about the treatment, saying: ‘Just when you think you know what pain is something comes along to show you some more. In hospital watching young Oshin getting sick and there is not a f***ing thing I can do about it.’”[20]
3. Rights of parents: much of the controversy centered on the challenging issue of parental rights versus the rights of the state. For example, in an interview with the Wernecke family, the interviewer Kerry Sanders stated, “Her father and mother argue this is a case of parental rights, but Texas state officials say those rights do not include the right to gamble with Katie [Wernicke]’s life.” [21] Regional differences exist in how each case was arbitrated, however, common among all cases was the theme of whether or not parents had the right to make a decision that they felt was in the best interest of the child if that decision involved abandoning a potentially curable treatment for an unproven one.
4. Evidence versus belief: also controversial was the right of parents to reject empirically based medical therapies for treatments they believed to be less harmful or more effective. Patients and parents sought a more personal and individual treatment then what they felt was available from the medical system. Cassandra Fortin, a 17-year old teenager diagnosed with Hodgkin lymphoma stated, “I am so sick of being treated like a number and how everything is based off of statistics. I am a patient not a number.” [22]
5. Rights of Indigenous Peoples: two cases, J. J. and Makayla Sault, both occurring in the same region of Canada, had the additional complexity of the Indigenous identity of the children involved. These controversial cases revealed the wide breadth of opinions held by Canadians with regard to the experience of Indigenous patients. For example, an editorial published in the *Calgary Herald* declared: “Traditions and community are wonderful things, but they are not cures for cancer. And if any indigenous treatments are potential cures, they should be used only after rigorous clinical trials have proven their efficacy, as happens with all other treatments.” [23] Conversely, an unattributed quotation from an article in the *National Post* noted, "Aboriginal healers, medicines pulled from the soil and even seers are fixtures of the community, located barely an hour southwest of Toronto, residents say. To many of the reserve's people, Western style medicine is alternative health-care and native remedies the mainstream, said one community leader.” [24]

**Table 2:** Themes and sub-themes identified, with example quotations.

Themes	Sub-themes
**Treatment as Torture**	a. Chemotherapy as poison *‴It's poison*,*'' Cassandra [Fortin]'s mother*, *Jackie Fortin*, *said of chemotherapy in an interview on Friday*. *''Does it kill the cancer*? *I guess they say it does kill the cancer*. *But it also kills everything else in your body*.*‴*[34] (Jackie Fortin, mother of Cassandra Fortin) b. Body as identity; violation of identity with forced treatments *“When I [Cassandra Fortin] woke up, I looked down and there was a port in my chest. I thought, 'I cannot believe they did this to me.' … 'I felt violated. I felt like I was being treated like an animal. I thought, "How can you force a person, regardless of how old they are, to do something they haven’t given consent to?”[35]* (Cassandra Fortin, teenager with lymphoma) c. Children traumatized by treatment *"He [Oshin Strachan] found it so difficult being in there, the procedures, he screamed and kicked and scratched. At a certain point they had to have six people hold him down and tie him with a sheet and have someone hold his head.”[36]* (Angela Kiszko, mother of Oshin Strachan) d. Fight or flight, violent resistance *“Until Tuesday, the family vehemently opposed chemotherapy on spiritual grounds, and Daniel [Hauser] had told a judge he would punch and kick anyone who tried to give it to him.” [37]* (Dan Linehan, reporter, about Daniel Hauser)
**Power Imbalances**	a. Capacity of children to make decisions *“Until they walk in his hospital slippers, they have no right to tread on his civil liberties where matters like this are concerned. Moreover, by age 11, a person is old enough to know what nausea, spinal pain, mouth sores, hair loss, being reduced to wearing diapers and having difficulty walking entail. … In this case, however, the choice appears to be possible recovery or certain death. It's an easy decision, but still not one a child can or should be expected to make.”[38]* (Letter to the editor about the case of Unnamed Child, 2008) b. Mistrust of physicians *“We like to believe that they’re going to do the best thing by us, but really, they’ve done the best thing by the pharmaceutical companies. Death by doctor is very common, but thankfully, because of the internet these days, a number of us have educated ourselves. There’s so many other options that we’ve been deprived of, denied. And it’s time for us all to wake up.”[39]* (Sally Roberts, mother of Neon Roberts) *“There’s dozens of things,” he [Abraham Cherrix] said, before launching into a discussion about medicinal mushrooms and the Rife ray machine. Both are alternative methods that have been denounced by the medical establishment. Alternative-medicine websites, though, tout them as methods “the medical Mafia” wants to keep under wraps.”[40]* (Abraham Cherrix, teenager with lymphoma) c. Perception of Bullying *“She [Aiden’s mother] got bullied in to agreeing to the chemo because she wanted to be with her kid. … I can understand why she did it.”[41]* (Marco Pederson, father of Aiden Pederson)
**Rights of Parents**	“Who gets to play God, to decide what’s best for a dying child? Should it be a doctor or should it be a parent?”[42] (Commentary about Oshin Strachan) a. Children as martyrs *“Adults are free to make martyrs of yourselves, but you can't make martyrs of your children. You can't kill your children in the name of your own religious beliefs.”[43]* (Jeffrey Toobin, legal analyst, about Jessica Crank) b. Perception of False Choices *“The couple [Brent Cassidy and Chloe O’Donohue] objects to a medical system that repeatedly asks for their consent over medical procedures but “reports” them when they refuse.” [44]* (Parents of Brayden O’Donohue) c. Parents offended by implication of being reported to child protective services *“He was happy the ordeal was over. "I never in a million years thought anyone would ever accuse me of not being a good father," he said.” [45]* (Jay Cherrix, father of Abraham Cherrix)
**Evidence versus Belief**	a. Misinformation/ Lack of understanding *“There is no evidence of cancer throughout her body*. *They just say radiation is here*, *and you have to do it*. *And they don’t give an explanation of why*. *You know*, *why treat a cancer that’s already dead*?*”* [46] (Edward Wernecke, father of Katie Wernecke) b. Rejection of scientific method *“They refused him chemotherapy and radiotherapy because they didn’t want their son to become a ‘lab rat’, the West Australian reports.” [20]* (Journalist comment about Oshin Strachan) c. Internet as information source *“Death by doctor is very common*, *but thankfully*, *because of the internet these days a number of us have educated ourselves… “There’s so many other options that we’ve been deprived of*, *denied*.*”* [39] (Sally Roberts, mother of Neon Roberts) d. Person versus patient *“Do what’s best for the patient*,*” said Conners*. *“Let’s take her case as an individual case and not try to fit her into what is standard protocol and look at her as an individual what she can tolerate what other therapies added to a traditional approach is going to be best for her*.”*[47]* (Dr. Kevin Conners, integrative cancer specialist, about Sarah Parisian) e. Alternative practitioners portrayed as villains *“Twice in the past six months, Clement [alternative practitioner] has visited Ontario First Nations communities, including Makayla's and J.J.'s, and given lectures on how his program teaches cancer sufferers to heal themselves. Clement makes his money telling desperate people exactly what they want to hear, regardless of empirical truth.” [48]* (Leah MacLaren, columnist, speaking about Makayla Smith and J.J.) f. Belief in alternative remedies as treatment *“Our belief is the natural stuff will do just as much as what that [chemotherapy] does if it's God's will.”[49]* (Andy Hershberger, father of Sarah Hershberger) g. Chemotherapy violating spiritual beliefs *“Daniel Hauser and his mother say using chemotherapy is against their spiritual beliefs, called Nemenhah. They are also Catholic. In place of chemotherapy, Colleen Hauser developed a regimen that stresses diet and includes supplements, herbs and water with a more acidic pH.” [50]*(Dan Linehan, reporter, about Daniel Hauser)
**Rights of Indigenous Peoples**	a. Political implications of decisions *“The band's defence of Makayla's decision also had political elements: an assertion of sovereignty and a rejection of history clouded by the residential schools' saga. … We're never going to allow another agency to ever do that to us again, where they remove our children from their community, from their culture, from their traditions," said Chief Bryan LaForme. "We are not going to let foreign governments come in and apprehend children.” [24]* (Chief Bryan LaForme, speaking about Makayla Sault) b. Concern about precedent set by legal decision *“… the result of his [Justice Gethin Edward’s] ruling is that every child in Canada is considered protected against the "wildly unreasonable decisions of their parents" except for First Nations children”* [51] (Arthur Schafor, ethicist, speaking about J.J.)

### Sentiment analysis

The SVM applied to the entire collected corpus of articles reported a 27% / 73% split between positive and negative articles. Most cases had an overall negative tone (See Table 3). Additionally, we looked at the positive or negative weight given to each word. For a given word, the weight corresponds to how important that feature is in generating a positive or negative prediction.

**Table 3:** Positive sentiment per case.

Case	Percentage of positive articles
Jessica Crank	0% (N = 33)
Abraham Cherrix	64% (N = 139)
Katie Wernecke	39% (N = 106)
Daniel Hauser	27% (N = 155)
Neon Roberts	27% (N = 156)
Sarah Hershberger	10% (N = 59)
Makayla Sault	13% (N = 113)
J.J.	22% (N = 62)
Cassandra Fortin	33% (N = 15)
Oshin Strachan	6% (N = 45)

The top 5 most positive and negative features from the training dataset are reported in Table 4. Precision reported from training is approximately 70%, compared with the precision reported by multiple humans classifying the same dataset has been reported as approximately 80%. [25] A review of a subset of the corpus resulted in a precision of 72%, which was expected from the training data.

**Table 4:** Most informative features extracted from the training dataset.

**Positive**	
**Word**	**Weight**
Family	1.74
Work	1.73
Use	1.61
Lung	1.51
Protest	1.50
**Negative**	
**Word**	**Weight**
Death	-2.13
Aboriginal	-1.72
Die	-1.71
Doctor	-1.48
Rate	-1.41

## Discussion

Media reports of children with cancer leaving CT for T&CM offer insight for health practitioners into public perception. From the 17 cases we identified, 5 major themes emerged: treatment as torture, power imbalances, rights of parents, evidence versus beliefs and rights of Indigenous Peoples. Tonality analysis of reports was largely negative, with the exception of reports about Abraham Cherrix (64% positive). It is unclear why this particular case had more positive coverage than other cases from the same time period (e.g. Katie Wernecke). This may be related to regional differences in reporting style and in acceptability of abandonment of therapy.

Standardized care, fundamentally responsible for the survival gains in pediatric cancer over the past half-century, was identified in several cases as a negative aspect of conventional care.[26] Families stated that they wanted their child to be treated as an individual as opposed to a “statistic”. Physicians as representatives of an industry that were “experimenting” on children was also a recurring theme. This sentiment is anathema to the vast majority of physicians; however, some families may enter the therapeutic relationship with profound mistrust. Working to include trusted T&CM practitioners in the care team may provide the approach to care that families require to build trust with the medical system.

In most countries, parents have a legal right to make decisions for their child; limitations exist on these rights to protect children from harm. This includes harm from failing to provide potentially curative medical therapy, known as medical neglect.[27] Controversy arises when the duty of the state to protect the child is pitted against opposing parental or individual rights. The case of Jessica Crank demonstrated conflict between the right to religious freedom and the State’s duty to protect children from medical neglect. Previous analyses have identified potential inadequacies in the laws in the United States protecting children from religiously motivated medical neglect.[28] The cases of J.J. and Makayla Sault set the rights of individuals as Indigenous Peoples to pursue Traditional medicine against the fiduciary duty of healthcare providers to protect children from harm.[29] The interplay between child protection rules as they pertain to medical responsibility and the rights of Indigenous families to choose Indigenous practices was described by ethicist Margaret Somerville as "a world of competing sorrows, because no matter what you do somebody is going to be hurt or harmed or upset".[30] In Canada, a long history of colonialism and post-colonial institutionalized racism has spawned significant mistrust between Indigenous patients and the healthcare and legal systems.[31, 32] Disturbingly, we found that the word “Aboriginal” was associated with a negative sentiment in news articles. As outlined in the *Calls to Action* of the Truth and Reconciliation Commission of Canada, it is imperative that healthcare practitioners recognize the importance of Traditional healing and incorporate these practices in the treatment of Indigenous patients where requested.[33]

Our study presents a mixed-methods approach to analyzing news reports in pediatric oncology. We used news media as primary source material to interrogate the data source most accessible to patients and families. Our approach allowed insight in to a larger corpus of published articles than would have been achievable with solely qualitative methods. Our study has several limitations. As is the case with all machine learning algorithms, the addition of more training data would improve the results of the automated sentiment analysis. Our study is also limited by the quality of source material available. Many important cases are likely kept out of the news media. Due to privacy laws in most countries, cases that are in the news media are brought forward by parents and families, and not hospitals or physicians. This is an important source of bias in any analysis of news media reports of a health topic. Further, LexisNexis and Factiva present only a sampling of the news available. Further methodological strategies are necessary with the advent of online news, and non-traditional news platforms such as social media.

We were unable to include information from low and low-middle income countries, where treatment abandonment is a more significant contributor to mortality, because of language barriers, availability of newspapers in online databases, and in settings where treatment abandonment is more common it is less likely to be “newsworthy”. News reports of abandonment of conventional cancer treatment for traditional and complementary medicine in low and low-middle income countries deserve careful scrutiny, however, it was outside the scope of this project. Further study should be focused on this area.

We present general assessments of overall tonality. Future analyses of media reports should attempt to quantify the positive or negative sentiment for specific elements, i.e. for or against providers, for or against parents, etc. This level of analysis was beyond this scope of this project. We noted that the term “doctor” was associated with a more negative sentiment, implying that there may be negative sentiment in news articles towards conventional health providers. Alternatively, “family” was associated with a positive sentiment. Future studies should consider interviewing families who have abandoned or attempted to abandon CT as a primary source. This task represents a daunting feasibility challenge, as the willingness of these families to participate in research may be lower than average.

We have analyzed news reports of families abandoning or attempting to abandon CT for T&CM. We hope a better understanding of what is portrayed to the public can help healthcare providers collaborate with families and T&CM providers to continue to improve outcomes for all children with cancer.

## Supporting information

S1 Appendix Search strategy. Search strategy for primary searches.
(DOCX)
