# Supplementary material for: ‘A world of competing sorrows’: A mixed methods analysis of media reports of children with cancer abandoning conventional treatment
Source: PLoS One. 2018 Dec 21;13(12):e0209738. doi: 10.1371/journal.pone.0209738 (PMC6303077; doi:10.1371/journal.pone.0209738)
Supplement: S1 Appendix — Search strategy for primary searches. (DOCX) [file pone.0209738.s001.docx]

**S1 Appendix: Search strategy.** Search strategy for primary searches.

**Factiva:**

Cancer AND (child or boy or girl) AND (Traditional medicine OR traditional healing OR Chinese medicine! OR Complementary medicine OR complementary therap! OR unconventional therap! OR Indigenous medicine! OR Aboriginal medicine! OR Folk medicine! OR shaman OR Homeopath! OR Naturopath! OR osteopath! OR acupunctur! OR chiropract! OR Traditional Chinese medicine! OR Faith healing OR ayurveda)

**LexisNexis:**

((Cancer AND child AND (Traditional medicine OR traditional healing OR Chinese medicine! OR Complementary medicine OR complementary therap! OR unconventional therap! OR Indigenous medicine! OR Aboriginal medicine! OR Folk medicine! OR shaman OR Homeopath! OR Naturopath! OR osteopath! OR acupunctur! OR chiropract! OR Traditional Chinese medicine! OR Faith healing OR ayurveda))
